# Plasmonic Properties of Individual Bismuth Nanoparticles

**DOI:** 10.1021/acs.jpclett.5c02531

**Published:** 2025-09-13

**Authors:** Michael Foltýn, Michal Kvapil, Tomáš Šikola, Michal Horák

**Affiliations:** † 613011 Brno University of Technology, Central European Institute of Technology, Purkyňova 123, Brno 612 00, Czech Republic; ‡ 48274 Brno University of Technology, Faculty of Mechanical Engineering, Institute of Physical Engineering, Technická 2, Brno 616 69, Czech Republic

## Abstract

Bismuth nanoparticles are being investigated due to their reported photothermal and photocatalytic properties. In this study, we synthesized spherical bismuth nanoparticles (50–600 nm) and investigated their structural and optical properties at the single-particle level using analytical transmission electron microscopy. Our experimental results, supported by numerical simulations, demonstrate that bismuth nanoparticles support localized surface plasmon resonances, which can be tuned from the near-infrared to the near-ultraviolet spectral region by changing the nanoparticle size. Furthermore, plasmonic resonances demonstrate stability across the entire spectral bandwidth, enhancing the attractiveness of bismuth nanoparticles for applications over a wide spectral range. Bismuth’s lower cost, biocompatibility, and oxidation resistance make bismuth nanoparticles a suitable candidate for utilization, particularly in large-scale and even industrial plasmonic applications.

## Graphical abstract

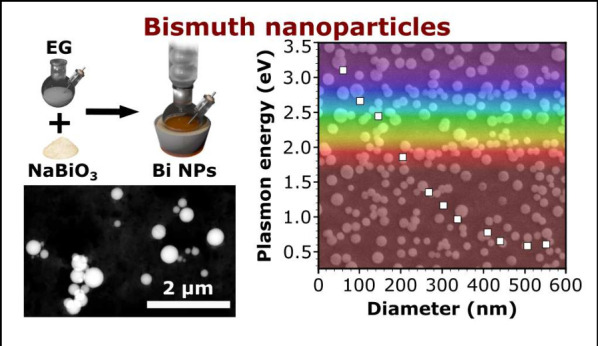

The biocompatibility, high atomic number, and accessible functionalization of bismuth nanoparticles make them a compelling candidate as a contrast enhancing agent in medical X-ray imaging and computational tomography techniques.^1,2^ Bismuth nanoparticles also exhibit quantum confinement effects,^3^ high Seebeck coefficients,^4^ and thermally driven semimetal to semiconductor transitions.^5^ Furthermore, their recently reported photocatalytic and photothermal properties also make them attractive for use in photothermal cancer therapies,^6,7^ environmental remediation,^8–10^ and energy storage.^11^ Theoretical studies have also predicted the ability of bismuth to support collective oscillations of free electrons, known as localized surface plasmon resonances (LSPR).^12,13^ These resonances enhance the local electromagnetic field in the vicinity of the nanoparticle, offering rich applications in biosensing,^14,15^ metasurfaces,^16^ and medicine.^17^ The potential combination of plasmonic applications and the extraordinary properties of bismuth has fueled research on bismuth nanowires^18,19^ and nanostructured bismuth thin films.^16,20–23^

However, studies on the plasmonic response of chemically synthesized bismuth nanoparticles have been limited only to investigations of plasmonic performance using far-field optical spectroscopy.^24,25^ The primary constraint of the method used in these papers was that it quantified the response of a large volume of solution containing nanoparticles of various sizes. Therefore, the recorded spectrum comprised contributions from nanoparticles of all sizes, resulting in an overlap of individual plasmonic resonances.^26^ Ultimately, in such an integral approach the dependence of the plasmon energy on the size of the nanoparticles cannot be determined and the plasmonic performance cannot be quantified.^27,28^ In order to obtain this information, an alternative analytical method with sufficient spatial and spectral resolution is required to measure the plasmonic resonances of individual nanoparticles. One of such a method is dark-field optical spectroscopy.^29^ However, in this method, nanoparticles must be laterally separated by distances larger than the diffraction limit and their scattering cross section high enough to give a sufficient detectable response.^30^ An useful alternative method is electron energy loss spectroscopy (EELS), an analytical technique frequently used in a scanning transmission electron microscope (STEM).^31^

The majority of wet nanoparticle syntheses rely on surfactants to prevent the aggregation and oxidation of synthesized nanoparticles. However, the surfactant molecules that are adsorbed onto the analyzed nanoparticles introduce an undesirable signal arising from their own excitations and alter the local dielectric environment.^32,33^ These detrimental effects have the potential to hinder EELS analysis or even prevent the extraction of signals emanating from plasmon resonances.^34^ It is imperative that a surfactant-free process should be employed during synthesis in order to successfully analyze the plasmonic response of individual bismuth nanoparticles by STEM EELS. Hence, in this study, we present the STEM EELS analysis of the optical response of individual single crystal bismuth spherical nanoparticles synthesized by a surfactant-free polyol process. The aim of this study is to explore the tunability of the dipole LSPR mode. In this study, we provide evidence that the dipole LSPR mode in bismuth nanoparticles can be tuned from the near-infrared (NIR) to the near-ultraviolet (UV) spectral region as a function of the nanoparticle diameter.

The schematic workflow for the synthesis and characterization of bismuth nanoparticles is shown in Figure 1. Bismuth nanoparticles were synthesized using a modified polyol process based on the synthesis approach reported in ref.^35^. The main steps of the reduction of sodium bismuthate in ethylene glycol at temperatures close to its boiling point are depicted in Figure 1a. For further details, see Methods. We note that we observed flocculation of nanoparticles in the solution after 1–2 days of standing. This phenomenon can be conceptualized as a reversible instability that can be readily broken down and subsequently restored employing ultrasound. Consequently, the solution can be regarded as sufficiently stable for utilization in plasmonics with regard to drop-casted nanoparticle applications. Next, we diluted the solution with methanol and dropped it onto commercially available SiO_2_ and carbon membranes for subsequent characterization by analytical transmission electron microscopy. The nanoparticles drop-casted onto a membrane are stable in air. Figure 1b shows the setup used for the STEM EELS analysis of synthesized nanoparticles. It includes a STEM annular dark field (ADF) image of a 107 nm nanoparticle, a background-subtracted electron energy loss spectrum integrated on the left edge of the nanoparticle, with a peak at 2.55 eV corresponding to the dipole LSPR mode, and an intensity map of electrons with an energy loss of 2.55 eV, the energy of the dipole mode.

**Figure 1:**
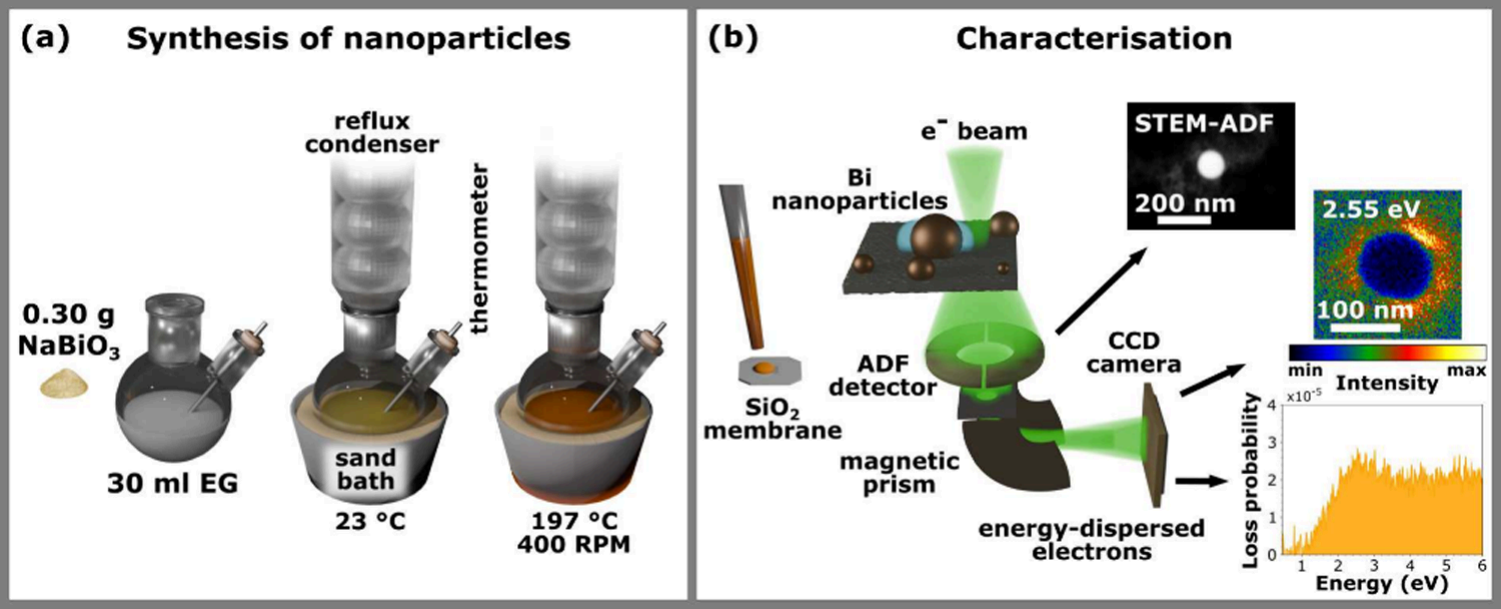
Schematic workflow of the synthesis and characterization of bismuth monocrystalline nanoparticles. (a) The nanoparticles were prepared by reducing sodium bismuthate in ethylene glycol at temperatures near its boiling point. (b) The morphology of the synthesized nanoparticles was obtained from STEM ADF micrographs and their plasmon resonances were examined using STEM EELS.

First, bismuth nanoparticles were inspected by analytical transmission electron microscopy to characterize their size, morphology, crystallinity, and chemical composition. Figure 2a shows a typical STEM high-angle annular dark-field (HAADF) micrograph of synthesized nanoparticles. The nanoparticles are generally spherical. However, smaller amounts of nanoparticles of different shapes, including nanowires, hexagonal nanoparticles, truncated nanotriangles, and nanosquares, were obtained, too (see Figure S1). The absence of surfactant molecules results in a rather wide-diameter distribution of the synthesized spherical nanoparticles. Based on the Gaussian fit of the diameter histogram, shown in Figure 2a, the average diameter of the nanoparticles is (137 ± 97), nm. We note that a narrower size distribution as well as preferential morphology of the nanoparticles can be adjusted by further tuning of the synthesis. In addition, the nanoparticles were sometimes embedded in layers of chemical residues (see Figure S2). Energy-dispersive X-ray spectroscopy (EDX) indicates that these chemical residues are likely unconsumed sodium bismuthate (see Figure S3). However, scanning the residue with the focused electron beam successfully removed them, likely through reduction induced by the electron beam. We note that the reduction of sodium bismuthate using an electron beam was reported in ref.^36^. Nevertheless, this does not preclude the utilization of the nanoparticles in environments devoid of an electron beam.

**Figure 2:**
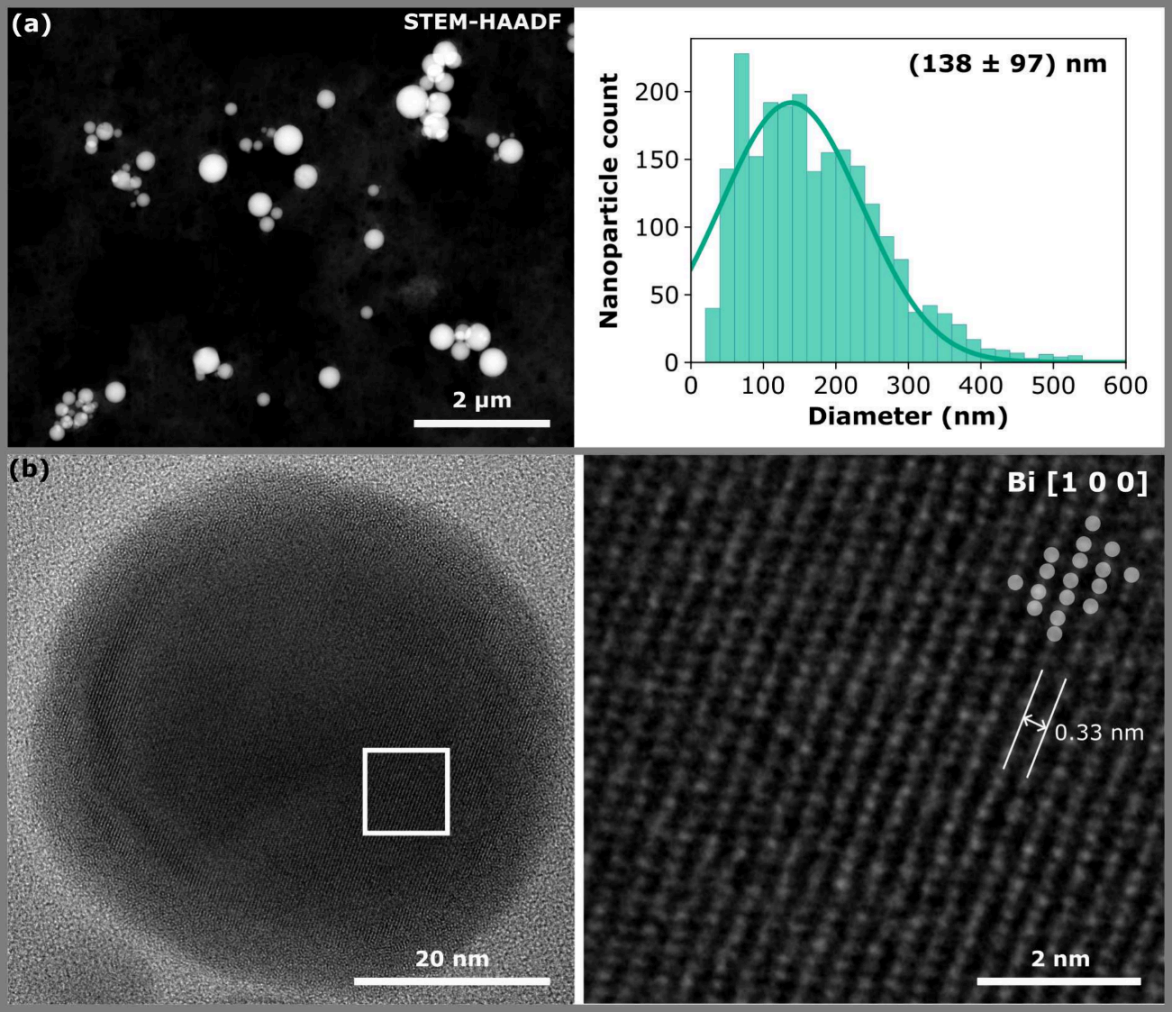
Morphology, size distribution and crystallinity of synthesized nanoparticles. (a) STEM HAADF micrograph showing their typical shape and size distributions. The nanoparticle diameter histogram diameter indicates large dispersion in size. (b) High-resolution TEM of a 54 nm nanoparticle showing that the nanoparticle is monocrystalline in [100] orientation. The white frame indicates the location of the zoomed area on the right.

Despite continuous growth during synthesis, the nanoparticles are monocrystalline. Figure 2b shows the high-resolution TEM micrograph of a 54 nm nanoparticle that demonstrates its monocrystallinity. Individual atomic columns are resolved. The image was further evaluated and compared with the crystalographic model of rhombohedral bismuth introduced in ref.^37^ using CrysTBox.^38^ As a result, the bismuth nanoparticle in the high-resolution TEM micrograph is in the [1 0 0] orientation with a distance of 0.33 nm between the atomic planes. In addition, there is no deep oxidation present at the nanoparticle surface despite the absence of surfactants in the synthesis. The surface consists of a native oxide layer that is just a few nanometres thick, which is further confirmed by the EDX shown in Figure S3. This is likely due to ethylene glycol and its low oxidation potential at elevated temperatures.^39^

Second, we have focused on the plasmonic properties of bismuth nanoparticles that are measured by STEM EELS. We have investigated a set of spherical nanoparticles with diameters ranging from 61 to 551 nm. The resulting processed EEL spectra are summarized in Figure 3a. Figure 3b shows the STEM HAADF micrographs of the nanoparticles with rectangles marking the integration areas from which the EEL spectra were collected. The spectra revealed pronounced plasmon peaks corresponding to the dipole mode present in all studied nanoparticles, ranging from 0.6 to 3.1 eV. The spectra are further fitted by Gaussians

$$g\left(x\right)=I_{\max}\cdot e^{-4\ln2\cdot {\left(\frac{x-E}{\Delta E}\right)}^{2}}$$

to obtain the characteristic parameters of the plasmon peaks, namely, the peak energy *E*, the loss probability maxima *I*_max_, and the full-width half-maximum (FWHM) *ΔE*. The results are summarized in Table 1.

**Figure 3:**
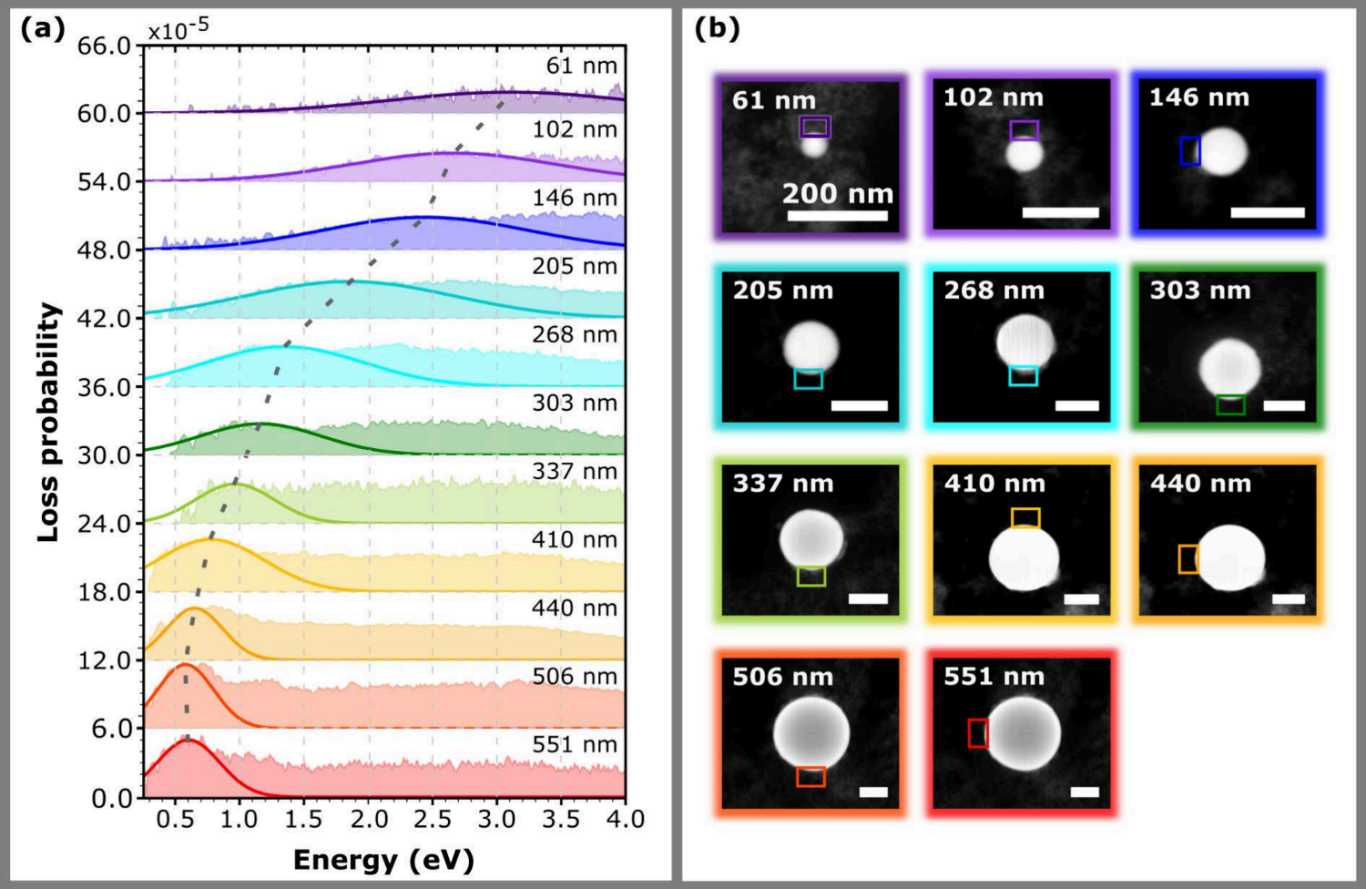
EELS analysis of spherical nanoparticles with diameter ranging from 61 to 551 nm. (a) Measured EEL spectra with the peaks corresponding to the dipole LSPR mode fitted with Gaussian. The dashed line is a guide for the eye that follows the dipole LSPR mode whose energy changes as a function of the particle diameter. (b) STEM HAADF micrographs of the nanoparticles with rectangles marking the areas from which the EELS signal was evaluated.

**Table 1:** Plasmonic Properties of Bismuth Nanoparticles

size (nm)	*E* (eV)	*I*_max_	*ΔE* (eV)	*Q* factor
61	3.10	1.9 × 10^–5^	0.95	3.3
102	2.66	2.6 × 10^–5^	0.80	3.3
146	2.44	2.9 × 10^–5^	0.79	3.1
205	1.86	3.2 × 10^–5^	0.81	2.3
268	1.35	3.5 × 10^–5^	0.61	2.2
303	1.16	2.8 × 10^–5^	0.47	2.5
337	0.96	3.5 × 10^–5^	0.30	3.2
410	0.78	4.6 × 10^–5^	0.41	1.9
440	0.65	4.6 × 10^–5^	0.22	3.0
506	0.58	5.6 × 10^–5^	0.22	2.7
551	0.60	5.0 × 10^–5^	0.24	2.5

The spectral tunability of the dipole plasmon mode by the nanoparticle diameter is graphically demonstrated in Figure 4a. Within the range of investigated nanoparticle diameters, the energy of the dipole mode covers the interval from 0.60 eV (corresponding to the wavelength of 2066 nm) for the 551 nm nanoparticle, to 3.10 eV (400 nm in wavelength) for the 61 nm nanoparticle. Therefore, bismuth nanoparticles represent a biocompatible and oxidation-resistant hyperspectral plasmonic platform that is tunable from the near-infrared to the near-ultraviolet part of the spectrum. Compared to other non-noble metals, this property is analogous to that of liquid and solid gallium^40,41^ or silver amalgam^42^ nanoparticles. Aluminum nanoparticles^43^ can be tuned from the visible to the ultraviolet and rhodium nanoparticles^44^ are tunable from the blue-visible to the ultraviolet part of the spectrum.

**Figure 4:**
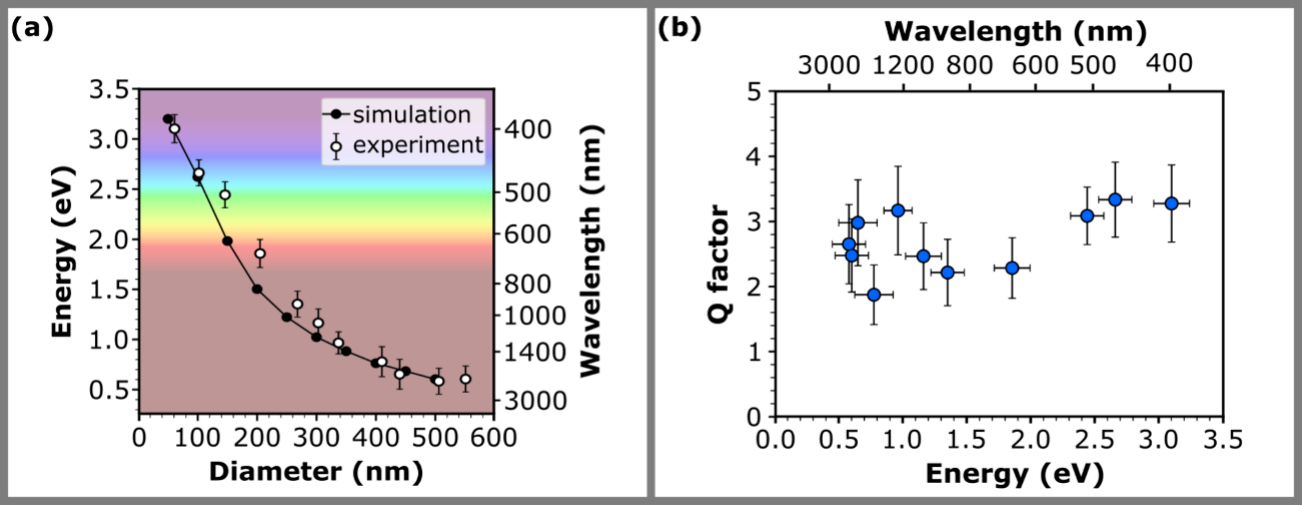
Spectral tunability of bismuth nanoparticles. (a) Dipole LSPR energy extracted from the measured and calculated EEL spectra as a function of nanoparticle diameter. (b) The *Q*-factors of the dipole plasmon mode extracted from the fits of the LSPR peaks in the measured EEL spectra.

Additionally, we have performed numerical simulations (see Methods) to support our experimental results. The calculated EEL spectra are shown in . The energy of the dipole mode, extracted from the simulations, is also shown in Figure 4a. We generally see a good agreement between the experiment and the numerical simulations that is further illustrated by the comparison of calculated and measured EEL spectra for the 200 nm bismuth nanospheres shown in . Small differences suggest a minor inaccuracy in the parameters used in the numerical model, including the exact shape of the nanoparticles, the dielectric function of bismuth, or the approximation of a silicon dioxide membrane by an effective surrounding medium.

The highest loss probabilities *I*_max_ of the dipole mode were observed in nanoparticles with diameters ranging from 410 to 551 nm, where the loss probability fluctuated around 5 × 10^–5^. For smaller nanoparticles, the loss probability decreases with the decreasing diameter of the nanoparticle, reaching the lowest loss probability of 1.9 × 10^–5^ for the 61 nm nanoparticle. The FWHM *ΔE* of the dipole LSPR mode exhibits the opposite trend. It increases with decreasing nanoparticle diameter and reaches 0.95 eV for the smallest (61 nm) nanoparticle and 0.24 eV for the largest (551 nm) nanoparticle, respectively. The Q factor (quality factor), defined as the LSPR energy divided by its FWHM, describes the sharpness of a resonance. A high Q factor is indicative of a narrow resonance line width, signifying that the plasmonic structure efficiently stores energy with minimal loss. The resulting Q factors range from 1.9 (for the 410 nm nanoparticle) to 3.3 (102 nm nanoparticle). They are shown in Figure 4b as a function of the energy of the dipole mode. When experimental uncertainty is taken into account, the Q factors are comparable for all nanoparticles analyzed, promising a comparable plasmonic performance over the entire available spectral range.

In conclusion, we have synthesized spherical monocrystalline bismuth nanoparticles from sodium bismuthate in ethyleneglycol through a solvothermal surfactant-free process. The nanoparticles were characterized using STEM-EELS to study their plasmonic properties at the single-particle level and to investigate the spectral tunability of localized surface plasmon resonances as a function of the nanoparticle diameter. The absence of surfactant molecules helped to avoid the influence of the EELS results on the locally different dielectric environment, enabling thus an unaffected study of the plasmonic response of individual nanoparticles. The results demonstrate that the absence of surfactants does not adversely affect the structure of the synthesized nanoparticles. The tunability of dipole plasmon resonant modes is provided by selecting the nanoparticle diameter, covering the interval from the near-infrared to the near-ultraviolet spectral region. Furthermore, plasmonic resonances show up stability across the entire spectral bandwidth, thereby enhancing the attractiveness of bismuth nanoparticles for applications over a wide spectral range and establishing them as a viable option for plasmonics. Moreover, the lower cost of bismuth, in conjunction with its biocompatibility and resistance to oxidation, renders it a suitable candidate for utilization, particularly in large-scale and industrial plasmonic applications.

## Methods

Monocrystalline bismuth nanoparticles were synthesized using a modified polyol process based on the synthesis approach reported in ref. 35. We dissolved 0.30 g of NaBiO_3_ in 30 mL of ethyleneglycol. We then placed the solution in a sand bath and gradually heated it to 197 °C while stirring at a rate of 400 rpm. When the temperature reached approximately 170 °C, the bright yellow solution turned dark orange. After maintaining the temperature of 197 °C for around 20 min, we switched off the heating and left the solution to slowly cool down to room temperature.

Analytical transmission electron microscopy investigation was performed on a TEM FEI Titan equipped with a GIF Quantum spectrometer. TEM measurements, including STEM HAADF imaging and atomic resolution TEM, were done at 300 keV. EELS measurements were carried out at 120 keV in the scanning monochromated mode with the convergence semiangle set to 10 mrad and the collection semiangle set to 11.4 mrad. The probe current was adjusted to around 100 pA. The dispersion of the spectrometer was set to 0.01 eV per channel and the FWHM of the zero-loss peak was around 0.15 eV. The acquisition time was adjusted to use the maximal intensity range of the CCD camera in the spectrometer and avoid its overexposure. These parameters were selected to acquire the EELS signal with the highest signal-to-background ratio.^45^ EEL spectra were integrated over rectangular areas at the edges of the nanostructures where the LSPR is significant. They were further divided by the integral intensity of the zero-loss peak to transform the measured counts into a quantity proportional to the loss probability. Next, the EEL spectrum of a pure silicon nitride membrane was subtracted to remove the background. Finally, the peaks corresponding to the dipole mode of LSPR were fitted by Gaussians.

Numerical simulations of EEL spectra were performed using the MNPBEM toolbox^46^ based on the boundary element method. Our model consisted of a bismuth sphere in an effective surrounding medium. The dielectric function of bismuth was taken from ref. 47 and the effective refractive index of the surrounding medium was set to 1.3 to approximate the effect of the silicon dioxide membrane substrate. The 120 keV electron beam was positioned 30 nm outside the nanoparticle.

## Supplementary Material





## Data Availability

Data sets for this manuscript are available in Zenodo at 10.5281/zenodo.15782716.

## Notes

STEM HAADF micrographs of bismuth nanoparticles capturing the occasional nonsperical shapes, the influence of the synthesis time on the resulting size of bismuth nanoparticles, contamination of the bismuth nanoparticles by residues imaged by STEM and analyzed by STEM EDX, and calculated EEL spectra of bismuth nanospheres (PDF)
Transparent Peer Review report available (PDF)
