# Supplementary material for: Plasmonic Properties of Individual Bismuth Nanoparticles
Source: J Phys Chem Lett. 2025 Sep 13;16(38):9933–8. doi: 10.1021/acs.jpclett.5c02531 (PMC12478859; doi:10.1021/acs.jpclett.5c02531)
Supplement: Supplementary file 1 [file jz5c02531_si_001.pdf]

Supporting Information: Plasmonic Properties of Individual Bismuth Nanoparticles

Michael Foltýn,[†] Michal Kvapil,[†,‡] Tomáš Šikola,[†,‡] and Michal Horák[*,†]

†*Brno University of Technology, Central European Institute of Technology, Purkyňova 123, Brno, 612 00, Czech Republic*

‡*Brno University of Technology, Faculty of Mechanical Engineering, Institute of Physical Engineering, Technická 2, Brno, 616 69, Czech Republic*

E-mail: michal.horak2@ceitec.vutbr.cz

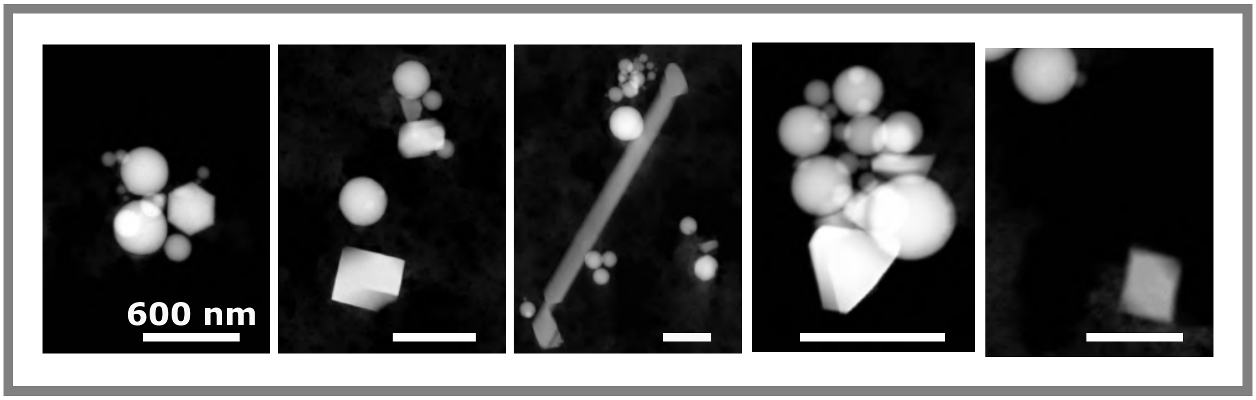

Figure S1: STEM HAADF micrographs of bismuth nanoparticles capturing the occasional nonsperical shapes like hexagonal, rod, triangular, or retangular nanoparticles. The scale bars are 600 nm long.

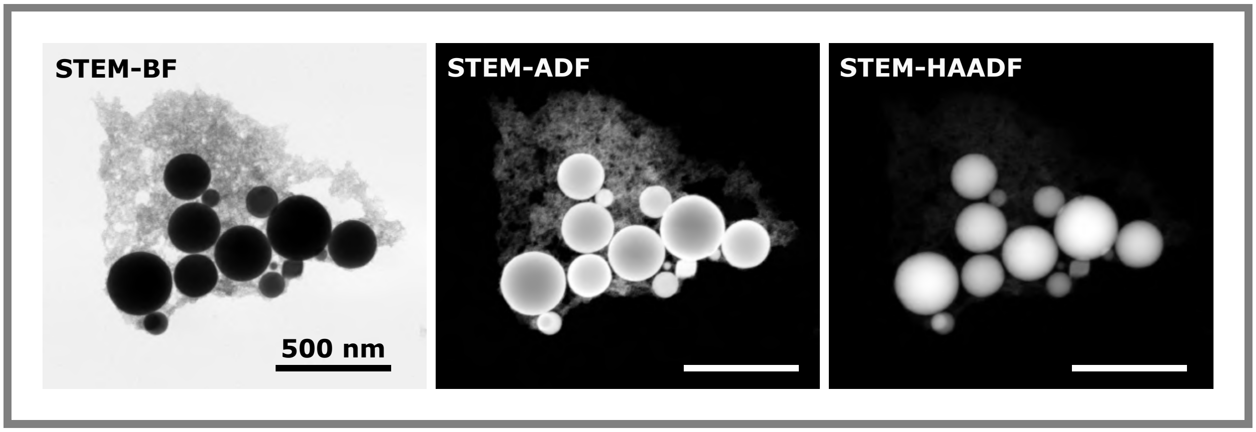

Figure S2: STEM imaging of bismuth nanoparticles dropcasted on a carbon TEM memebrane using different detectors. STEM bright field (BF) and annular dark field (ADF) captures the diffraction contrast, whereas STEM high-angle annular dark field (HAADF) signal is dominated by Z-contrast or mass contrast so thin or light-element composed structures may become invisible. Bismuth nanoparticles are sometimes embedded in layers of chemical residues that due to low effective mass are clearly visible in STEM BF, but almost invisible in STEM HAADF.

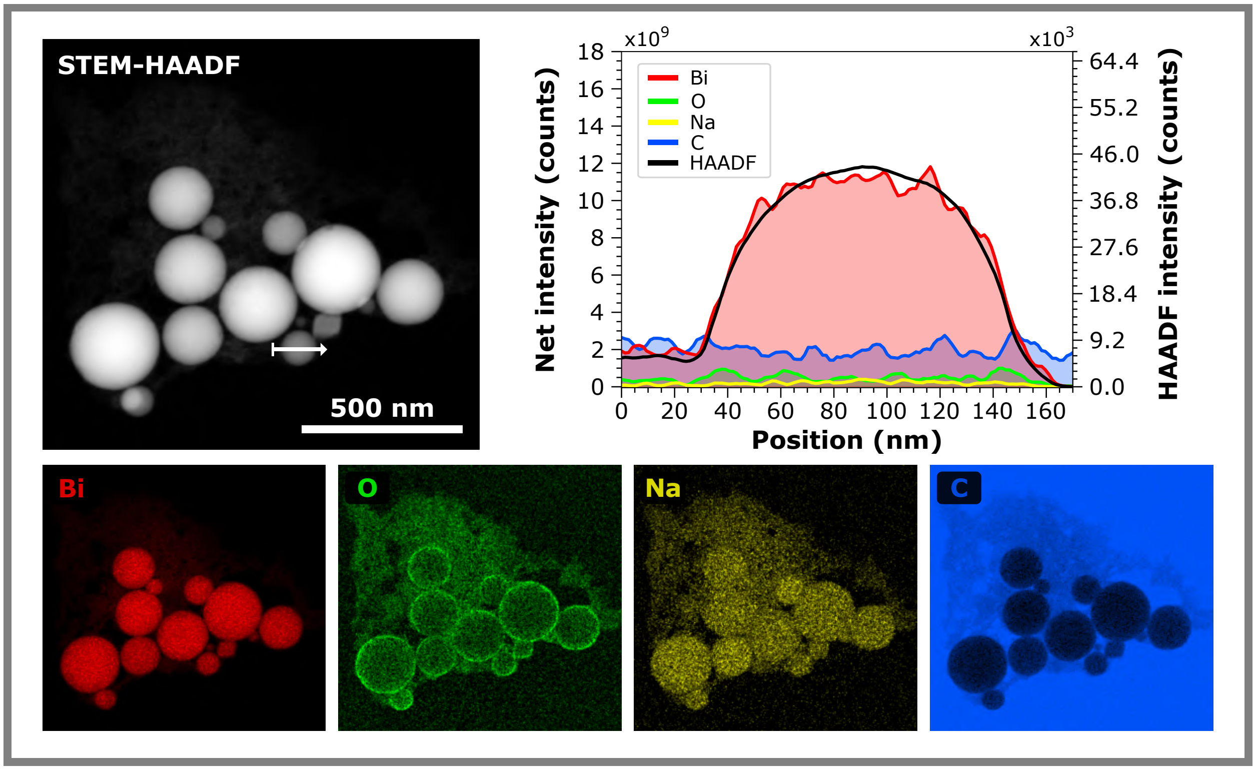

Figure S3: Material analysis of the bismuth nanoparticles, shown in Figure S2, performed by STEM EDX (energy-dispersive X-ray spectroscopy) mapping. Net intensity maps of bismuth, oxygen, sodium, and carbon are shown. The carbon signal comes primary from the carbon membrane. The layer of chemical residues corresponds to unconsumed $NaBiO_3$.

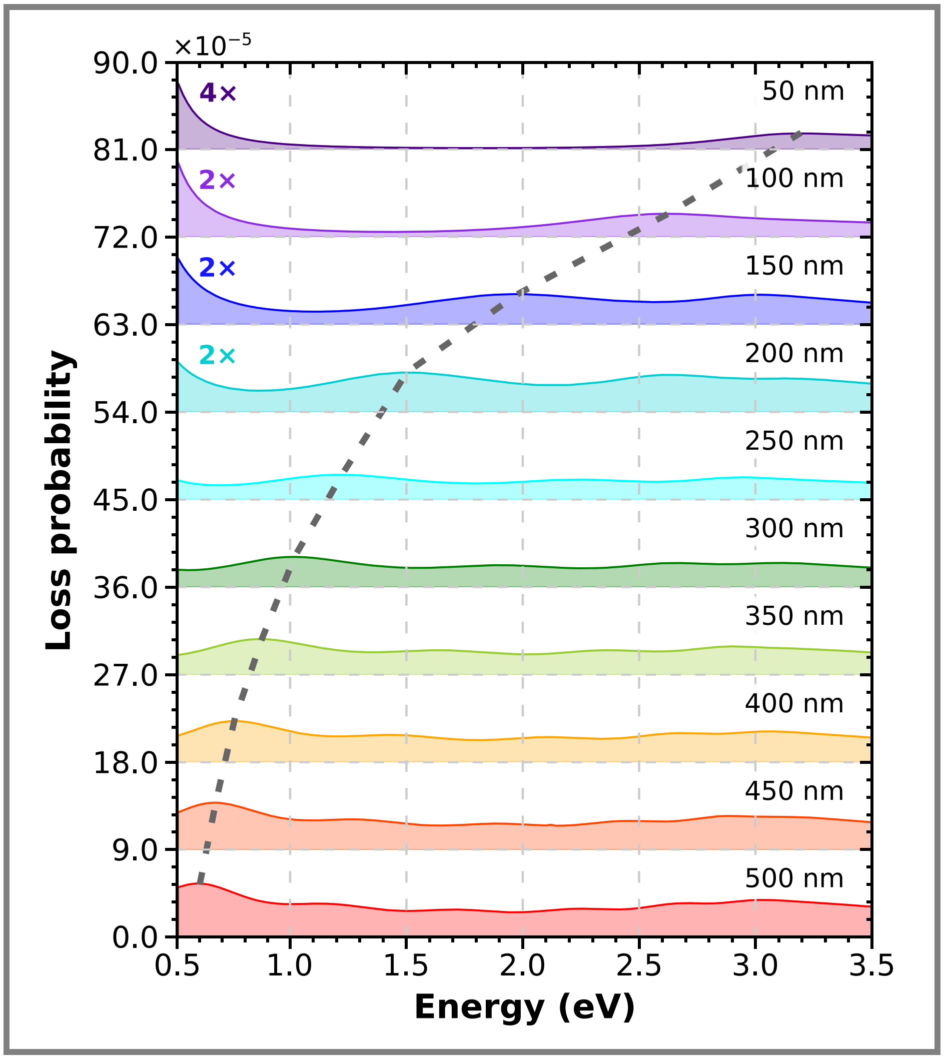

Figure S4: Numerical simulation of EEL spectra of bismuth nanospheres with the diameters in the range from 50 nm to 500 nm. The electron beam is 30 nm outside the nanosphere. The dashed line is intended to serve as a guide for the eye, following the peaks corresponding to the dipole LSPR mode.

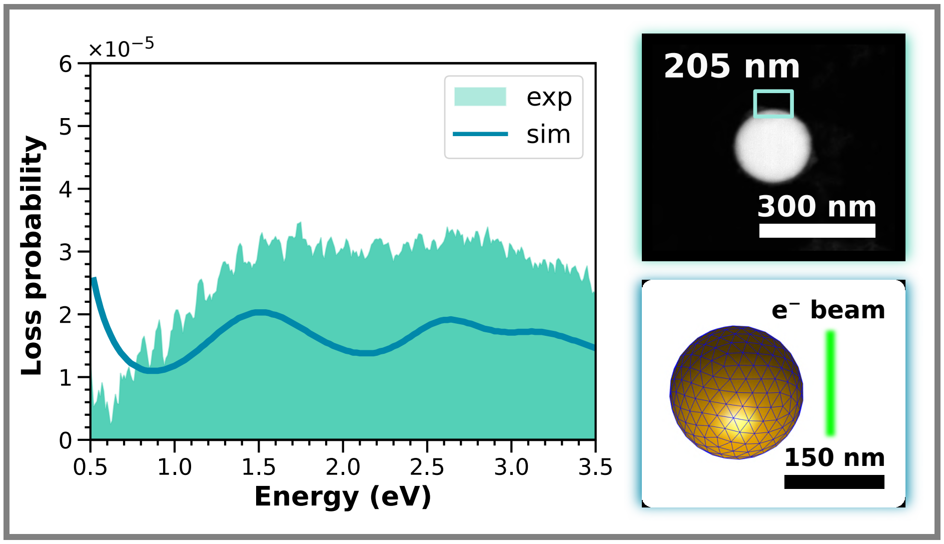

Figure S5: Comparison of calculated and measured EEL spectra for the 200 nm (numerical simulation) and 205 nm (STEM EELS experiment) bismuth nanospheres. Calculated and measured EEL spectra are in a good agreement. The higher loss probability in the experiment is caused by a minor discrepancy in the electron beam position between the experiment (integrated over the marked rectangular area at the edge of the nanoparticle) and the simulation (electron beam is situated 30 nm outside the nanosphere).
