# Supplementary material for: Plasmonic Properties of Individual Bismuth Nanoparticles
Source: J Phys Chem Lett. 2025 Sep 13;16(38):9933–8. doi: 10.1021/acs.jpclett.5c02531 (PMC12478859; doi:10.1021/acs.jpclett.5c02531)
Supplement: Supplementary file 2 [file jz5c02531_si_002.pdf]

jz-2025-02531p.R1

Name: Peer Review Information for "Plasmonic Properties of Individual Bismuth Nanoparticles"

First Round of Reviewer Comments

Reviewer: 1

Comments to the Author

In this work, these authors synthesized spherical bismuth nanoparticles (50-600 nm). They demonstrated that bismuth nanoparticles support localized surface plasmon resonances, which can be tuned from the near-infrared to the near-ultraviolet spectral region by changing the nanoparticle size. Furthermore, plasmonic resonances demonstrate stability across the entire spectral bandwidth, enhancing the attractiveness of bismuth nanoparticles for applications over a wide spectral range. But the following questions should be addressed and answered before that article is published:

1. Please provide relevant experimental data to prove the stability of bismuth nanoparticles synthesized by a surfactant-free polyol process.

2. The author should provide relevant formulas or references to prove how the highest loss probabilities of the bismuth nanoparticle dipole mode were calculated.

3. The author states in the manuscript that “scanning bismuth nanoparticles with the focused electron beam successfully removed the chemical residues (sodium bismuthate) surrounding them, likely through reduction induced by the electron beam.” Does this indicate that the bismuth nanoparticles synthesized by this method are not suitable for use in environments without the electron beam?

4. What does the loss probability of the dipole mode affect? Is it the distance of light propagation? Please briefly explain in your manuscript.

5. The morphology and particle size of the bismuth nanoparticles synthesized by the author using the synthetic method employed in the study were uneven. How to balance the

relationship between the morphology, particle size of bismuth nanoparticles, and the amount of surfactant used in the application process?

Reviewer: 2

Comments to the Author

The reviewed manuscript, jz-2025-02531p, presents a comprehensive theoretical and experimental investigation of bismuth nanoparticles. The main contribution lies in the detailed analysis of their optical and structural properties at the single-particle level, employing analytical transmission electron microscopy. The experimental findings are corroborated by numerical simulations, enhancing the robustness of the study. The work provides novel experimental data that are likely to be of significant interest to the readership of the Journal of Physical Chemistry Letters and experts who deal with nanomaterials. Accordingly, I recommend the manuscript for publication in the Journal of Physical Chemistry Letters.

Comments: I recommend that the authors explicitly articulate the aim of the study at the beginning of the manuscript.

Author's Response to Peer Review Comments:

Ing. Michal Horák, Ph.D.
Brno University of Technology, CEITEC
Purkyňova 123, 612 00 Brno, Czech Republic
michal.horak2@ceitec.vutbr.cz

September 4, 2025

Response to the Decision Letter

Dear Editor,

we thank you for handling our manuscript and the Reviewers for the comments, which we thoroughly utilized to improve the manuscript, and we are glad to submit the Revised Manuscript article entitled “Plasmonic Properties of Individual Bismuth Nanoparticles” by Michael Foltýn, Michal Kvapil, Tomáš Šikola, and Michal Horák for consideration for publication in *The Journal of Physical Chemistry Letters.*

The full response to the Reviewers' comments follows at the end of this document. Further, the manuscript PDF with all changes made is uploaded as the Supplementary material for Review only, too.

This manuscript is currently not under consideration for publication elsewhere.

The preprint version of this manuscript is published on Arxiv [http://arxiv.org/abs/2506.16248].

The datasets for this manuscript will be freely available in Zenodo under DOI 10.5281/zenodo.15782716 as soon as the manuscript is accepted [http://doi.org/10.5281/zenodo.15782716].

We have no conflicts of interest to disclose.

We thank you for the care that you take of our manuscript.

Sincerely, Michal Horák on behalf of all Authors

Response to the Reviewers' comments and additional changes

We thank the Reviewers for their very useful comments, which we thoroughly utilized to improve the manuscript. A point-by-point response to the Editorial and Reviewers' comments follows. The Reviewer’s report is in blue, our comments in black, and the corresponding action in green.

Reviewer: 1

Recommendation: This paper is probably publishable, but major revision is needed; I do not need to see future revisions.

Comments:

In this work, these authors synthesized spherical bismuth nanoparticles (50-600 nm). They demonstrated that bismuth nanoparticles support localized surface plasmon resonances, which can be tuned from the nearinfrared to the near-ultraviolet spectral region by changing the nanoparticle size. Furthermore, plasmonic resonances demonstrate stability across the entire spectral bandwidth, enhancing the attractiveness of bismuth nanoparticles for applications over a wide spectral range. But the following questions should be addressed and answered before that article is published:

We thank Reviewer 1 for the evaluation of our manuscript. In the following, we address the individual points.

1. Please provide relevant experimental data to prove the stability of bismuth nanoparticles synthesized by a surfactant-free polyol process.

The zeta potential was not measured in order to verify the stability of the solution. The observation of flocculation of nanoparticles in solution was made after 1-2 days of standing. This phenomenon can be conceptualized as a reversible instability that can be readily broken down and subsequently restored, employing ultrasound as a representative example. Flocculation is not ordinarily a problematic aspect unless the solution is to be utilized directly in an application. Consequently, given the reversibility of this flocculation, the solution can be regarded as sufficiently stable for utilization in plasmonics with regard to drop-casted nanoparticle applications. The nanoparticles drop-casted onto a membrane are stable in air.

We have added this discussion to the manuscript. The updated part reads as follows:

"…Bismuth nanoparticles were synthesised using a modified polyol process based on the synthesis approach reported in Ref. [10.1021/jp063474e]. The main steps of the reduction of sodium bismuthate in ethylene glycol at temperatures close to its boiling point are depicted in Figure 1a. For further details, see Methods. We note that we observed flocculation of nanoparticles in the solution after 1-2 days of standing. This phenomenon can be conceptualized as a reversible instability that can be readily broken down and subsequently restored employing ultrasound. Consequently, the solution can be regarded as sufficiently stable for utilization in plasmonics with regard to drop-casted nanoparticle applications. Next, we diluted the solution with methanol and dropped it onto commercially available SiO_2 *and carbon membranes for subsequent characterisation by analytical transmission electron microscopy. The nanoparticles drop-casted onto a membrane are stable in air. Figure 1b shows…"*

2. The author should provide relevant formulas or references to prove how the highest loss probabilities of the bismuth nanoparticle dipole mode were calculated.

The loss probability maxima were obtained from the Gaussian fitting. We added the formula for the Gaussian into the corresponding text in the manuscript. It was not included before, as we supposed that the formula for the Gaussian function is well-known.

The updated part reads as follows:

"The spectra are further fitted by Gaussians

$$g(x) = I_{max} \cdot \mathrm{e}^{-4\ln 2\left(\frac{x-E}{\Delta E}\right)^2}$$

to obtain the characteristic parameters of the plasmon peaks, namely, the peak energy E, the loss probability maxima I_{max}, and the full-width half-maximum (FWHM) ΔE. The results are summarised in Table 1."

3. The author states in the manuscript that "scanning bismuth nanoparticles with the focused electron beam successfully removed the chemical residues (sodium bismuthate) surrounding them, likely through reduction induced by the electron beam." Does this indicate that the bismuth nanoparticles synthesized by this method are not suitable for use in environments without the electron beam?

It means that some of the dropcasted nanoparticles were embedded in layers of chemical residues. These residues can be removed by an electron beam. There are different ways to remove the residues from the solution, such as repeated centrifugation and re-dispersion of the created pellet in a suitable solvent. In addition, the presence of these residues does not significantly affect the plasmonic properties of bismuth nanoparticles – just a small energy shift is expected due to a small change of dielectric environment around the nanoparticle. A similar energy shift is expected when a different substrate is used. Consequently, nanoparticles synthesized by this method are perfectly suitable for use also in environments without the electron beam.

We have added a note to the manuscript. The updated part reads as follows:

"In addition, the nanoparticles were sometimes embedded in layers of chemical residues (see Figure S2). Energy-dispersive X-ray spectroscopy (EDX) indicates that these chemical residues are likely unconsumed sodium bismuthate (see Figure S3). However, scanning the residue with the focused electron beam successfully removed them, likely through reduction induced by the electron beam. We note that the reduction of sodium bismuthate using an electron beam was reported in Ref. [10.1088/0957-4484/18/33/335604]. Nevertheless, this does not preclude the utilization of the nanoparticles in environments devoid of an electron beam."

4. What does the loss probability of the dipole mode affect? Is it the distance of light propagation? Please briefly explain in your manuscript.

The loss probability I_{max} of the dipole mode is defined as the peak height of the Gaussian function that is fitted to the corresponding peak in the electron energy loss spectra. The term "loss probability" is employed to denote the likelihood that an incident electron will lose a specific amount of energy due to inelastic scattering. In this particular instance, this corresponds to the excitation of the dipole mode of LSPR. Consequently, it has no connection to any distance of light propagation. It just refers to the magnitude of the dipole LSPR mode.

For better clarity, we include the symbols I_{max} and □E in the corresponding text. The updated part reads as follows:

"The highest loss probabilities I_{max} of the dipole mode…"

"The FWHM □E of the dipole LSPR mode…"

5. The morphology and particle size of the bismuth nanoparticles synthesized by the author using the synthetic method employed in the study were uneven. How to balance the relationship between the morphology, particle size of bismuth nanoparticles, and the amount of surfactant used in the application process?

This is a very good comment. In our study, we target the tunability of bismuth nanoparticles; therefore, the non-uniform size of the synthesized nanoparticles is advantageous for us. We did not aim for a uniform (narrow) size distribution. Similarly, we did not target nonspherical shapes. This will be a subject of further research. Generally, the size distribution of the nanoparticles and their shapes are influenced by the concentration of reactants, temperature, reaction time, as well as possible surfactants.

We have added a note to the manuscript. The updated part reads as follows:

"The nanoparticles are generally spherical. However, smaller amounts of nanoparticles of different shapes, including nanowires, hexagonal nanoparticles, truncated nanotriangles, and nanosquares, were obtained, too (see Figure S1). The absence of surfactant molecules results in a rather wide-diameter distribution of the synthesised spherical nanoparticles. Based on the Gaussian fit of the diameter histogram, shown in Figure 2a, the average diameter of the nanoparticles is (137 □ 97) nm. We note that a narrower size distribution as well as preferential morphology of the nanoparticles can be adjusted by further tuning of the synthesis."

Additional Questions:

Urgency: High
Significance: Top 10%
Novelty: High
Scholarly Presentation: Top 10%
Is the paper likely to interest a substantial number of physical chemists, not just specialists working in the authors' area of research?: Yes

Reviewer: 2

Recommendation: This paper represents a significant new contribution and should be published as is.

Comments:

The reviewed manuscript, jz-2025-02531p, presents a comprehensive theoretical and experimental investigation of bismuth nanoparticles. The main contribution lies in the detailed analysis of their optical and structural properties at the single-particle level, employing analytical transmission electron

microscopy. The experimental findings are corroborated by numerical simulations, enhancing the robustness of the study. The work provides novel experimental data that are likely to be of significant interest to the readership of the Journal of Physical Chemistry Letters and experts who deal with nanomaterials. Accordingly, I recommend the manuscript for publication in the Journal of Physical Chemistry Letters.

We thank Reviewer 2 for the evaluation of our manuscript.

Comments: I recommend that the authors explicitly articulate the aim of the study at the beginning of the manuscript.

We have updated the end of the third paragraph as follows:

"…Hence, in this study, we present the STEM EELS analysis of the optical response of individual singlecrystal bismuth spherical nanoparticles synthesised by a surfactant-free polyol process. The aim of this study is to explore the tunability of the dipole LSPR mode. In this study, we provide evidence that the dipole LSPR mode in bismuth nanoparticles can be tuned from the near-infrared (NIR) to the near-ultraviolet (UV) spectral region as a function of the nanoparticle diameter."

Additional Questions:

Urgency: High
Significance: High
Novelty: High
Scholarly Presentation: High
Is the paper likely to interest a substantial number of physical chemists, not just specialists working in the authors' area of research?: Yes

Additional changes – updates in References:

We updated the Reference to the Preprint

~~(22) Foltýn, M.; Šikola, T.; Horák, M. Bismuth plasmonic antennas. 2025; https://arxiv.org/abs/2504.00671, Accessed: August 13, 2025.~~

to the published manuscript (published online on September 1, 2025)

(22) Foltýn, M.; Šikola, T.; Horák, M. Bismuth plasmonic antennas. ACS Nano **2025**,

We note that volume, issue, and pages are not assigned by the publisher at the time of revising this manuscript. Other information, such as doi, is included in the BibTeX bibliography file.

Next, we updated the References to the gallium nanoparticles in the discussion by adding a recently published manuscript (published online on August 21, 2025; no citable preprint available before):

"…Compared to other non-noble metals, this property is analogous to that of liquid and solid gallium[40,41] or silver amalgam[42] nanoparticles."

(41) Horák, M.; Foltýn, M.; Čalkovský, V.; Mikerásek, V.; Bartošík, M.; Mach, J.; Šikola, T. Plasmonic Response to Liquid–Solid Phase Transition in Individual Gallium Nanoparticles. The Journal of Physical Chemistry Letters **2025**, *16*, 8891–8896.
